# Contemporary Considerations in the Evolution of Wearable Technology for Arrhythmia Detection

**DOI:** 10.2174/1573403X19666230811093048

**Published:** 2023-10-02

**Authors:** Tobin Joseph, Mahmoud Barrie, Akbar Karimi, Sharmi Haque, Innocent Ogunmwonyi, Utkarsh Ojha

**Affiliations:** 1 Department of Acute Medicine, Hillingdon Hospital, Uxbridge, United Kingdom;; 2 School of Medicine, Imperial College London, London, United Kingdom;; 3 Faculty of Health, Medicine and Life Sciences, Maastricht University, Maastricht, Netherlands;; 4 Department of Medicine, Darent Valley Hospital, Dartford, Kent, United Kingdom;; 5 Chelsea and Westminster Hospital, London, United Kingdom;; 6 Royal Brompton and Harefield Hospital, Harefield Hospital, London, United Kingdom

**Keywords:** Arrhythmia, monitoring, wearables, technology, cardiovascular complications, cardiac devices

## Abstract

Arrhythmias are an increasingly common cause of hospital admissions worldwide. Late detection of arrhythmias is associated with a significantly increased risk of cardiovascular complications. Early identification and management of life-threatening arrhythmias is paramount to reduce mortality. Wearable technologies are now widespread among the general population, providing a continuous output of healthcare data. However, this data are not routinely integrated into clinical practice. Here, we begin by outlining the current landscape in wearable technology for aiding arrhythmia detection; we then consider the clinical impact of wearable technology for both clinicians and patients; we further highlight the latest and emerging trials in wearable technology for arrhythmia detection and finally postulate the wider implications of the expansion of such cardiac devices.

## 1 INTRODUCTION

An arrhythmia is a deviation from the normal heart rhythm caused by aberrancy within the electrical conduction system [1]. Within the general population, the prevalence of arrhythmias is estimated to range from 0.96% for those below the age of 55 to 4.84% for patients aged over 65 [2]. The current global trend of increasing average population age and comorbidity means that physicians worldwide will likely see an increase in the prevalence of arrhythmias [2, 3]. Arrhythmias remain a common cause of hospital admission, most prevalent among elderly patients or those with comorbidities [2].

Arrhythmias can remain inconspicuous for many years, sometimes detected in patients incidentally. In other patients, arrhythmias can lead to immediate symptoms, including shortness of breath, palpitations, and syncope [1, 3] Modern diagnostic techniques employ physical examination and electrophysiological studies to characterise arrhythmias, using many features, including heart rate and regularity. This form of assessment allows recognition of the arrhythmia and its nidus, which can guide further management [4]. Prolonged untreated arrhythmias are associated with a significantly increased risk of cardiovascular complications, including stroke, heart failure and sudden death [5].

Arrhythmias also place a significant financial burden on healthcare systems. Economic analysis suggests that in 2020 alone, atrial fibrillation (AF) cost the National Health Service (NHS) up to £2.5 billion - with most of the expense derived from primary admissions. In the next two decades, AF is projected to use up to 4.27% of the total NHS expenditure [6]. Consequently, early detection and management of arrhythmias are essential for reducing both the public health burden and financial pressures on healthcare systems.

Given the acceleration of technological innovation within recent years, it stands to reason that wearable devices may eventually be able to accurately and efficiently detect arrhythmias. Herein we evaluate the current state of wearable devices in arrhythmia detection and highlight the progress within emerging trials. Furthermore, we consider this technology's impact on patients, clinicians, and healthcare systems overall.

## 2 CURRENT CHALLENGES IN EVALUATING ARRHYTHMIA

### 2.1 Non-invasive Investigations can have a Low Diagnostic Yield

An electrocardiogram (ECG) remains the gold standard for assessing heart rhythm, where the output records the heart’s electrical activity at a specific point in time. However, this snapshot is often insufficient to detect a rhythm disturbance, especially in those suffering from paroxysmal disease. The European Society of Cardiology (ESC) recommends various modalities for optimizing arrhythmia detection and management [3, 4].

Holter monitoring is an ambulatory ECG device capable of monitoring cardiac activity for an extended time period. However, 24-hour Holter monitors have a low diagnostic yield (between 15 – 39%), principally because many patients are asymptomatic in the days succeeding hospital admission and that, by definition, arrhythmias are paroxysmal in nature [7].

### 2.2 Invasive Investigations have Higher Risks

Further investigations include an implantable loop recorder (ILR). This small subcutaneous device is capable of recording ECG snapshots of tachyarrhythmias or bradyarrhythmias and recording activity for up to 3 years [8]. ILRs do improve the diagnostic yield for arrhythmias, with studies citing diagnoses in up to 60% of patients [9, 10]. Although effective for long-term monitoring, its invasive implementation has some risks, including bruising, discomfort and infection. Though having a high initial cost, early cost-effectiveness analyses demonstrated that in certain cohorts of patients, ILRs are of benefit [11, 12].

### 2.3 Decisions Regarding Anticoagulation

Current evidence suggests once an atrial arrhythmia is detected, anticoagulation is commenced to minimise the risk of stroke. This must be counter-balanced with the risk of bleeding, and clinicians must factor in age, frailty, and tendency to fall, amongst other factors, when deciding on the introduction of anticoagulation. Often, given the paroxysmal nature of most arrhythmias, these patients can be placed on lifelong anticoagulation from the time it is first detected.

However, emerging research suggests that early anticoagulation may not improve the risk of stroke or cardiovascular morbidity when continuous monitoring for AF is performed with ILRs [13]. This evidence further lends itself to the idea that careful monitoring of patients could be useful. However, given the low diagnostic yield of non-invasive investigations, it can be difficult to quantify when the arrhythmia burden increases enough to cross a risk threshold.

An affordable, simple, non-invasive device capable of accurate, continuous cardiac monitoring would be ideal to successfully identify arrhythmias without requiring patients to undertake an invasive procedure. Ongoing research is needed whether continuous monitoring would mitigate the need for early therapeutic anticoagulation.

### 2.4 The Current Landscape of Wearable Technology

There has been a rapid progression in the functionality of wearable electronic devices, which currently feature a high degree of adaptability and convenience. The integration of wireless communication systems with these devices has allowed them to become a regular part of life within the general population [14]. Smartwatches and smart wristbands are the most popular smart devices. Current trends suggest a significant growth in the number of wearable devices used each year, increasing from 325 million in 2016 to 722 million in 2019. Over one billion devices are projected to be in use [14, 15]. Although wearable devices have traditionally been associated with a younger population, there has been a steady increase in their use among the elderly population due to their increased ease of use and capabilities [16].

Currently, wearable technologies can track heart rate, and some include an irregular heartbeat notification. This can lead to the presentation of an asymptomatic individual to a healthcare provider. Alternatively, patients often can try to time their symptomatology to heartbeat variations detected by their smart device and use this to ask for further investigation. These technologies are broadly in two categories.

### 2.5 Electrocardiography

There are two methods of measuring heart rate and rhythm analysis using wearable technology [17]. The first method involves wearable electrocardiograms, which measure electrical potential across the chest. Several electrodes are placed around the chest, depending on whether the patient has an ECG or Holter monitor. ECGs classically have 12 leads which are derived by analysing the different electrical signals between the 10 different electrodes. The machine then interprets these measurements to produce graphs demonstrating the relative changes in electrical current between certain electrodes. These are then computationally analysed to produce the ECG strips that each provide a certain view of the heart [18]. This underpins the concept of 24-hour tapes, ILRs and other ‘traditional’ wearable technology pieces – by reducing the number of worn electrodes, the number of recorded leads can be reduced to compromise between diagnostic yield and patient comfort.

The use of ECG is well-established, relatively cheap, and clinically valid. However, each ECG-based method has significant limitations–notwithstanding the burden for the patient, inconvenience and the risks of invasive procedures [14, 17]. A further significant drawback is that these technologies are normally used reactively i.e., it is used in the detection of arrhythmia in patients who may exhibit associated signs and symptoms, giving a higher positive predictive value (such as syncope) [17].

**Fig. (1):**
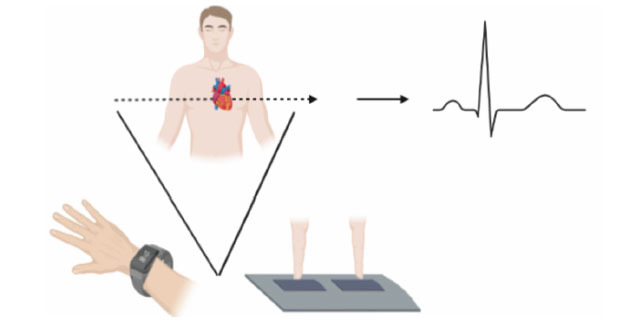
Demonstration of ECG technology and its utilisation in wearable technology. Electrical signals are taken from two separate points on the body to generate a rhythm strip, commonly equating to lead II. On watches (bottom left), this often requires the sensor on the wrist plus a finger on the contralateral hand placed on the watch to generate a rhythm strip. Other devices can use fingers from each hand to generate a rhythm strip (bottom right).

In newer generations of wearable technology, this technique is further miniaturised by producing one lead from the ECG by using the corresponding 2 electrodes. Classically, this is lead II on a 12-lead ECG. By reducing the number of captured signals from 12 to 1, the technology can be miniaturised with the two different electrodes being on the face of a watch (Fig. **1**).

### 2.6 Photoplethysmography

**Fig. (2):**
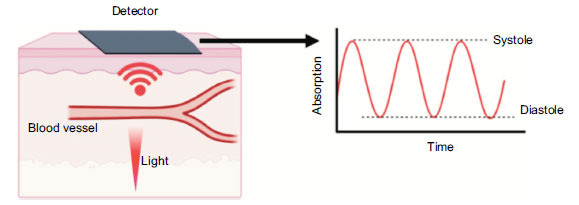
Demonstration of photoplethysmography and the generation of a waveform.

The second method for arrhythmia detection is photoplethysmography (PPG). [17] This term describes the method of measuring light that is scattered by blood flow once shone onto the skin. This is done by shining light (typically green light due to its depth of penetration) with a photodetector on the opposite side, *e.g*., on a fingertip or around a wrist. The photodetector then calculates the amount of light absorption to determine the oxygen saturation. Heart rate is calculated by the variation in pulsatility during light detection [19], [20]. This data is used in conjunction with other components, such as accelerometers, which detect the motion of the subject. Algorithms then integrate this data into motion-tolerant heart rate data but can also calculate additional biometrics such as calories burned, R-R interval, heart rate variability, blood metabolite concentrations, blood oxygen levels, and even blood pressure (Fig. **2**) [19, 20].

A light is shone through a blood vessel, and the amount of absorption is detected on a sensor on the opposite side of the vessel. When the heart is in systole, vessels dilate to accommodate blood flow leading to greater absorption. Arterial recoil leads to less absorption, leading to a waveform. The peaks on the waveform can be counted to calculate a heart rate.

When utilised in wearable technology, the diode emits light towards a vessel and measures the reflected light back. This is then calculated, with variations in absorption being attributed to the pulsatile nature of the artery being measured. This allows the diode and sensor to be in one device, *e.g.,* on the back of a smartwatch or smart bracelet.

There are an increasing number of FDA-approved devices for arrhythmia monitoring. A recent summary of the current wearable devices for physiological monitoring has been published, including their FDA approval status [21].

### 2.7 Implications for Clinicians

Accurate detection, symptom correlation and monitoring efficacy of treatment can all be seen as major hurdles for the physician when investigating arrhythmias. Current monitoring methods such as Holter devices and ILRs can impact the quality of patient care. This is especially important when one considers the rising tide of consumer-grade wearables that can be used for arrhythmia detection, which can be much more convenient for the patient [17, 21].

This has been exemplified in studies such as the REHEARSE-AF study – a randomised controlled trial that compared standard care to the use of the AliveCor Kardia monitor for 1001 patients. The study demonstrated that bi-weekly screening in ambulatory patients using the AliveCor is significantly more likely to detect AF [5]. A study by Seshadri *et al*. assessed the utility of the Apple Watch in cardiac patients who were already on telemetry. The notification based system only had a 41% sensitivity for AF (which was confirmed using telemetry), whereas the saved PDFs of rhythm strips yielded a 96% sensitivity – suggesting that notifications stating that an arrhythmia is present may be erroneous but manual interpretation of PDFs may be of diagnostic relevance [22]. Further research is needed to assess the impact of the other commercially available devices, and caution should be taken before actioning reports from these commercial devices.

Currently, the ESC recommends that a 30-second single lead rhythm strip interpreted by a physician can be used to diagnose AF [3]. This is in contrast to the National Institute of Clinical Excellence (NICE) guideline, which still recommends manual palpation followed by 12 lead ECG and then prolonged monitoring with a Holter [23]. Though there is a need for larger-scale studies into the diagnosis of arrhythmias, AF being the most common globally, it seems logical that a blended approach suggested by the ESC seems the most logical.

### 2.8 Evaluating Arrhythmias other than AF

This area will become more complex when arrhythmias other than AF are considered, such as ventricular tachycardia or bradyarrhythmias. There have been case reports published of bradycardia, higher-grade atrioventricular block and ventricular tachycardia being noticed on a wearable device [24-26]. There currently is no published evidence on the use of wearable technology outside the detection of AF. This is likely due to the prevalence of AF and its known association with patient morbidity and mortality.

The recently published eBRAVE-AF trial demonstrated increased rates of AF detection for those being digitally screened using certified apps on their smartphones, findings which were confirmed with a 14-day holter. The trial found a significant difference in the detection rate of AF in the cohort that were being digitally screened [27]. This is corroborated by the recently published Fibit Heart Study monitored 455,699 patients using Fitbit devices, and found that those with an irregular heartbeat notification were likely to have AF which was confirmed on an ECG patch after an initial notification. The positive predictive value was calculated to be 98.2% [28].

### 2.9 Monitoring and Screening

There is currently both a paucity of data and limited evidence regarding the use of wearable technology for the detection and management of arrhythmias. Advancements in wearables' clinical validity would lead toward more targeted and personalised therapeutic options. In the context of atrial fibrillation, there is the potential to move from a discrete phenomenon to a more continuous, burden-defined management where antiarrhythmics, rate control and anticoagulation decisions are made based on the burden of arrhythmia. There is also the possibility that in post-myocardial infarction, the single lead ECG could be used to screen for the burden of atrial or ventricular arrhythmias and thus can lead to earlier up-titration of medication [29].

This would greatly aid clinicians in guiding medication management and monitoring the success of interventions such as cardioversion and ablation. A recent consensus statement by the ESC and European Heart Rhythm Association (EHRA) called for further studies on the role of wearables in the monitoring of chronic cardiac conditions [30]. These studies will also assist in the validation of ECG and PPG technologies for arrhythmia detection as increasingly larger numbers of patients are recruited into these trials. Though there are several FDA-approved devices, the number of total devices is far greater than those with FDA approval.

### 2.10 Emerging Evidence of Wearable Technology

For the use of wearables in screening for arrhythmia, there is conflicting evidence. The recent LOOP study demonstrated that though there was an increased rate of detection for AF in patients with an ILR, there was no significant difference in outcomes [13].

There are multiple ongoing trials assessing the utility of wearables in screening for arrhythmia. These are mainly for the investigation of AF due to its prevalence and association with morbidity and mortality.

The SAFER Wearables Study (A Study of the Acceptability and Performance of Wearables for Atrial Fibrillation Screening in Older Adults) is a case-control study where patients will be asked to wear three devices for three days (two ECG and one PPG monitoring device) followed by a questionnaire on their experiences. The study will involve patients over 65, either with or without AF [31].

The REMOTE-AF (Remote Monitoring of AF Recurrence using mHealth Technology) is an observational cohort study. It aims to compare a wearable device with an ILR to detect the recurrence of AF in patients that have already undergone an ablation. This will be using a PPG-based device, and will be in patients over the age of 18 [32].

The HEARTLINE study (A Heart Health Study Using Digital Technology to Investigate if Early AF Diagnosis Reduces the Risk of Thromboembolic Events Like Stroke In the Real-world Environment) is an observational cohort study aiming to enroll 28000 patients over the age of 65. It aims to monitor whether patients using an Apple Watch (Series 5 or later) and an iPhone will engage with an app designed to engage patients with cardiovascular health. It will also assess whether the app impacts the concordance with anticoagulation [33].

The Artesia Trial (Apixaban for the Reduction of Thrombo-Embolism in Patients With Device-Detected Sub-Clinical Atrial Fibrillation) is an event-driven trial where patients are randomised to receive either Apixaban or aspirin in patients with subclinical atrial fibrillation detected by either a pacemaker, implantable cardiac defribillator (ICD) or ILR in patients over the age of 55 [34].

These ongoing trials demonstrate the potential use and integration of wearables in the diagnosis, management and surveillance of arrhythmias. This includes potentially increasing patient ownership of their health conditions and also can help clinicians when making decisions regarding medication for symptom burden and prognostication. The results of these trials are eagerly anticipated as they will help incorporate the use of these technologies within future guidelines.

## 3 IMPLICATIONS FOR PATIENTS

### 3.1 Improved Patient Concordance

The role of wearable technology in patients’ lives has increased their awareness and anecdotally has increased their awareness of their own health. Wang *et al*. demonstrated that in patients with AF, they tend to engage more with their healthcare providers though this increased engagement did not change their rate control significantly [35]. Other studies have also demonstrated that increased patient engagement can help reduce disease burden [36].

App-based measurements could also measure patient engagement with their healthcare – tracking medication and anticoagulation concordance. This enables healthcare providers to intensify intervention towards patients who are not concordant with medical treatment to improve disease burden. Wearable technologies could also enable direct communication between patients and their healthcare providers, as patients could use the downloaded rhythm strips from continuous monitoring to demonstrate a paroxysmal arrhythmia to their clinician [37]. The experience could also be used to reinforce positive health behaviours. [36] This increased engagement with their health condition can also lead to increased concordance with therapies, as they could get real-time feedback on the burden of their arrhythmia. Increased engagement is crucial to driving overall health costs and disease burden down, and it can often be underestimated in the care for a patient [36].

Acceptability and uptake of wearables in elderly patients, who are generally deemed the higher risk cohort for arrhythmias, vary, which is further compounded in the usability and comfort. Amongst this patient group, confidence and physical activity awareness often increase uptake [36].

### 3.2 Earlier Diagnosis of Arrhythmias or Change in their Nature

One key implication is that wearables can aid in the earlier diagnosis of arrhythmias. This in turn could have a significant impact on the morbidity and mortality of these arrhythmias, though there is little evidence at present. The potential impact of wearable technologies could revolutionise clinical medicine. In the field of arrhythmias, it can be used to detect the burden of the arrhythmia and whether there is any clinical change in the nature of the arrhythmia.

However with this constant monitoring of data, patients must be wary of the availability of their healthcare data and clinicians must be increasingly aware of the ethical implications of continuously monitoring their patients [38].

### 3.3 Driving

Driving is a key experience of patient lives, and arrhythmias can drastically alter a patient's relationship with driving. Wearable technology could help with the diagnosis of an arrhythmia that leads to an accident, or refute a cardiogenic diagnosis when medical practitioners are assessing a patient's suitability to drive. [39] This is increasingly important when considering patients that drive heavy goods vehicles – who are known to be at increased cardiovascular risk due to the disruptive lifestyle. [40] Continuous monitoring with wearable technology could enable closer monitoring of higher risk groups (once the technology is validated). Theoretically, it could also minimise misdiagnosis or unnecessarily labelling patients as unfit to drive.

### 3.4 Wider Implications

The wearable technology sector is expected to reach a market valuation of $392 billion by 2030 at a compound annual growth rate (CAGR) of 13.89% [41]. This rapid expansion of the industry will undoubtedly create competition between companies to manufacture more novel devices. The increased competition between manufacturers could subsequently result in newer devices not receiving the same level of scrutiny and regulations. Currently, wearable devices which are intended for diagnostic purposes must receive clearance from regulatory bodies such as the US Food and drug administration (FDA) and European Medicines Agency (EMA). However, some companies may change their marketing strategies leading to legal ambiguities which could exempt their devices from receiving clearance from regulatory bodies. Moreover, cardiac devices such as ICDs and pacemakers have previously been targets for hackers [42]. A report by Muddy Waters Research LLC in 2016 described the risk posed to cardiac devices manufactured by St Jude Medical. The report outlined the possibility of hackers causing the devices to increase the pacing rate and drain battery power [43]. As wearable cardiac technologies become more widespread, patients have understandably raised concerns regarding the risk of hacking and misuse of private patient data [44]. Despite the growing popularity of wearable devices, it is pertinent that the regulatory landscape continues to evolve to ensure the devices reaching the market maintain the highest level of safety and accuracy.

## CONCLUSION

Wearable technologies are an emerging field, and their utility within the field of cardiology is rapidly expanding. With this rapid expansion, however, there is the need for more evidence to determine the efficacy, feasibility and safety of using these technologies in the detection and management of arrhythmias. There are multiple ongoing trials that will provide further evidence on how best to integrate the use of wearable technology in clinical medicine. When considering the increased uptake of wearables in those at most risk, such as older patients, wearable technology holds the potential to be a non-invasive, reliable tool for the detection and optimisation of management of patients' arrhythmias.

## LIST OF ABBREVIATIONS

ECG: Electrocardiogram
ESC: European Society of Cardiology
ILR: Implantable Loop Recorder
CAGR: Compound Annual Growth Rate
FDA: Food and Drug Administration
